# Antioxidant and Anti-Fatigue Constituents of Okra

**DOI:** 10.3390/nu7105435

**Published:** 2015-10-26

**Authors:** Fangbo Xia, Yu Zhong, Mengqiu Li, Qi Chang, Yonghong Liao, Xinmin Liu, Ruile Pan

**Affiliations:** Institute of Medicinal Plant Development, Peking Union Medical College, Chinese Academy of Medical Sciences, No 151, North Road Malianwa, Haidian District, Beijing 100193, China; xiafb08@163.com (F.X.); xiaoyu.880609@163.com (Y.Z.); 13821058176@126.com (M.L.); qchang@implad.ac.cn (Q.C.); yhliao@implad.ac.cn (Y.L.)

**Keywords:** okra, anti-fatigue, antioxidant, polyphenols

## Abstract

Okra (*Abelmoschus esculentus* (L.) Moench), a healthy vegetable, is widely spread in tropical and subtropical areas. Previous studies have proven that okra pods possess anti-fatigue activity, and the aim of this research is to clarify the anti-fatigue constituents. To achieve this, we divided okra pods (OPD) into seeds (OSD) and skins (OSK), and compared the contents of total polysaccharides, total polyphenols, total flavonoids, isoquercitrin, and quercetin-3-O-gentiobiose and the antioxidant activity *in vitro* and anti-fatigue activity *in vivo* between *OSD* and *OSK*. The contents of total polyphenols and total polysaccharides were 29.5% and 14.8% in OSD and 1.25% and 43.1% in OSK, respectively. Total flavonoids, isoquercitrin and quercetin-3-O-gentiobiose (5.35%, 2.067% and 2.741%, respectively) were only detected in OSD. Antioxidant assays, including 1-diphenyl-2-picrylhydrazyl (DPPH) scavenging, ferric reducing antioxidant power (FRAP) and reducing power test, and weight-loaded swimming test showed OSD possessed significant antioxidant and anti-fatigue effects. Moreover, biochemical determination revealed that that anti-fatigue activity of OSD is caused by reducing the levels of blood lactic acid (BLA) and urea nitrogen (BUN), enhancing hepatic glycogen storage and promoting antioxidant ability by lowering malondialdehyde (MDA) level and increasing superoxide dismutase (SOD) and glutathione peroxidase (GSH-PX) levels. These results proved okra seeds were the anti-fatigue part of okra pods and polyphenols and flavonoids were active constituents.

## 1. Introduction

Fatigue is a complex physiological phenomenon, which is defined as difficulty in initiating or sustaining voluntary activities [1]. Apart from increasing with age and presenting in cancer, depression, HIV infection, multiple sclerosis and Parkinson’s disease patients, fatigue is becoming a more and more common symptom in normal humans with the increasing pace of modern life [2,3,4]. Large community surveys have showed that more than half of the adult population complains about fatigue [5,6]. Long-term accumulated or chronic fatigue not only lowers the quality of life, but also leads to chronic-fatigue syndrome and other organic illnesses, and even leads to *karoshi* (the abnormal death from acute cardiovascular diseases caused by overwork [7]). There are several theories about the causes of fatigue, such as exhaustion theory, which states fatigue is caused by a lack of available ATP for actin-myosin coupling, Na^+^/K^+^ pumping and Ca^2+^ uptake by the sarcoplasmic reticulum [8,9,10,11]; homeostasis disturbance theory, which indicates fatigue is caused by the accumulation of various metabolic byproducts [10,12]; and catastrophe theory indicating that fatigue may develop due to failure at one or several sites along the pathway of force production [13]. Among these, oxidative stress is widely thought to play an important role in the etiology of fatigue [10,13,14,15,16]. Antioxidants from some herbs have been shown to possess considerable anti-fatigue effects, which indicates that antioxidant treatment might be a valuable anti-fatigue therapeutic approach [17,18,19,20]. Therefore, consumers are seeking more natural antioxidant components in their diet to reduce oxidative damage and fight against fatigue.

Okra (*Abelmoschus esculentus* (L.) Moench, Family: *Malvaceae*), also known as lady’s finger, bhindi and gumbo, is an annual plant native to Africa and has been grown in different countries around the world, mainly in tropical, subtropical and warm temperate regions. The pods of okra have long been used as a vegetable and a source of dietary medicines. In traditional Asian and African medicine, okra pods are used as a mucilaginous food to combat gastritis [21]. Pharmacological studiess have revealed that okra possesses antioxidant, neuroprotective, antidiabetic, antihyperlipidemic, and anti-fatigue activities [22,23,24,25,26].

Although the anti-fatigue activity of okra pods has been proven before [26], little is known about its anti-fatigue activities and constituents. Previous literature has reported that okra pods contain high contents of polysaccharides, polyphenols and flavonoids [27,28,29,30,31,32,33,34]. It has also been demonstrated that polyphenols and flavonoids possess strong antioxidant and anti-fatigue effects in previous studies [15,18,20,35,36]. What is more, our preliminary qualitative experiment, in which we used chloride ferric solution as a color developing reagent, revealed that okra seed extract displayed a strong phenolic reaction; however, its skin extract hardly displayed such reaction (Supplementary materials), which demonstrated that okra seeds contained higher contents of polyphenols and flavonoids than okra skins. Therefore, it could be speculated that okra seeds may be the anti-fatigue part of okra pods, and polyphenols and flavonoids may be the active constituents.

The aim of the study was to clarify the anti-fatigue constituents of okra pods. To achieve this, we divided the fresh okra pods into seeds and skins, and compared the chemical contents of total polysaccharides, total polyphenols, total flavonoids, isoquercitrin and quercetin-3-O-gentiobiose, and antioxidant activities *in vitro* and anti-fatigue activities *in vivo* between okra seeds and skins. All these results revealed that okra seeds were the anti-fatigue part of okra pods, and polyphenols and flavonoids were active constituents. The potential mechanisms of the anti-fatigue activities in okra were also studied.

## 2. Experimental Section

### 2.1. Plant Material and Extraction

The fresh okra pods were purchased from a market (Sanya, Hainan Province, China) in July 2013. The plant was authenticated by Professor Bengang Zhang, the Institute of Medicinal Plant, Chinese Academy of Medical Sciences and Peking Union Medical College, Beijing, China, where the voucher specimens (No. 20130705) have been deposited in the institute’s Herbarium.

Two portions of 2.5 Kg fresh okra pods were prepared. One portion was lyophilized directly to obtain dried okra pods (251.3 g). The other portion was divided into okra seeds and okra skins, lyophilized to obtain, respectively, dried okra seeds (50.1 g) and dried okra skins (200.5 g). The okra pods, seeds, and skins were well ground and extracted separately with 1500 mL boiling water each for 1 h (3 times). Each filtered liquid was combined and concentrated under vacuum, to yield residues of okra pods (OPD, 105.4 g), okra seeds (OSD, 20.5 g) and okra skins (OSK, 84.1 g), respectively. Therefore, the ration of extract of OPD:OSK:OSD is about 5:4:1. All samples were stored at −20 °C until subsequent chemical analysis and animal experiments.

### 2.2. Chemicals and Reagents

Isoquercitrin and quercetin-3-O-gentiobiose were isolated from okra pods by our laboratory, at purities are over 98%, by high performance liquid chromatography (HPLC) analysis. Rutin, Gallic acid and D-glucose standards were purchased from National Institute for Food and Drug Control (Beijing, China). Folin–Ciocalteu’s phenol reagent (1N, Coolaber Science & Techinology, Beijing, China) was used for the determination of phenols. Ferric reducing antioxidant power (FRAP) kit was purchased from Beyotime Biotechnology (Beijing, China). The 1,1-diphenyl-2-picrylhydrazyl (DPPH) and trolox were purchased from Sigma-Aldrich Inc. (St. Louis, MO, USA). Phosphate buffer (PBS, Gibco) was purchased from Thermo Fisher (Waltham, MA, USA). The solvents, including acetonitrile (Thermo Fisher, MA, USA) and formic acid (CNW, Dusseldorf, Germany), were HPLC grade, and water was obtained from a Milli-Q water purification system (Millipore, MA, USA). Assay kits for determination of blood lactate (BLA), serum urea nitrogen (BUN), hepatic glycogen (HG), glutathione peroxidase (GSH-PX), thiobarbituric acid (TBA) and superoxide dismutase (SOD) were purchased from Nanjing Jiancheng Biotechnology Institute (Nanjing, China). All other reagents and chemicals used in this study were of analytical grade.

### 2.3. Animals

Male ICR mice (20–22 g) were purchased from the Vital River Laboratories (Qualified No.: SCXK 2012-0001, Beijing, China). The mice were housed in groups of 6 animals per cage under a constant temperature (23 °C ± 2 °C) and humidity (50% ± 10%) on a 12/12-h light/dark cycle (lights on from 8:00 a.m. to 8:00 p.m.). The animals had free access to standard chow diet and sterilized drinking water in the SPF (Specific pathogen Free) animal house. All experimental procedures were conducted under the supervision and approval of the Academy of Experimental Animal Center of the Institute of Medicinal Plant Development and in strict accordance with the NIH Guide for the Care and Use of Laboratory Animals (Eighth edition)[37].

### 2.4. Chemical Analysis of OSD and OSK

#### 2.4.1. Determination of Total Flavonoid (TF) Content

The TF content was measured by NaNO_2_-Al(NO)_3_ colorimetric assay according to the Technical Standards For Testing and Assessment of Functional Food, which was issued by National Health and Family Planning Commission of the PRC in 2003, with minor modifications. Briefly, 50 mg sample was dissolved in 40 mL distilled water and sample solution was extracted by 50 mL chloroform three times. Then, the water solution was diluted to 50 mL and 2 mL of the diluted solution was adsorbed by 5 g polyamide resin and eluted with 20 mL 70% ethanol. The 70% ethanol elution was concentrated to 10 mL under vacuum. Three milliliters of concentrated elution was placed in a 10 mL volumetric flask, and then 2 mL 70% ethanol, 0.3 mL NaNO_2_ (5%) and 0.3 mL Al(NO)_3_ were added. After 5 min, 2 mL NaOH (1.0 M) solution was added to the mixture solution and allowed to stand for 6 min. Finally, 2.4 mL 30% ethanol was added. The absorbance was measured against a blank at 510 nm with a UNICO-2012 UV-VIS spectrophotometer (Shanghai, China). The total flavonoid content was calculated from the calibration curve using rutin as the standard (20–100 mg/L). The results were reported as mg of rutin equivalent per mg of samples.

#### 2.4.2. Determination of Total Polyphenol (TP) Content

The TP content was determined using Folin–Ciocaiteu method with slight modification [38]. In short, 50 mg sample was mixed with 25 mL of 50% methanol solution, then 0.5 mL sample solution, 0.3 mL Folin–Ciocaiteu’s reagent and 10 mL sodium carbonate (10%) were sufficiently mixed, and then the volume was adjusted to 25 mL with distilled water. The mixture was allowed to stand at 50 °C in darkness for 1 h. Absorbance was measured at 765 nm. A calibration curve of Gallic acid was prepared. The results were expressed as mg of Gallic acid equivalents per mg of samples.

#### 2.4.3. Determination of Isoquercitrin and Quercetin-3-O-Gentiobiose

Quantification of isoquercitrin and quercetin-3-O-gentiobiose was performed by HPLC-UV using a five-point calibration curve (*r*^2^ = 0.999) in the range of 5–500 μg/mL. Isoquercitrin and quercetin-3-O-gentiobiose (5 mg, respectively) and samples (10 mg) were dissolved in 10 mL methanol, respectively. All solutions were filtered through 0.45-μm polytetrafluoroethylene filters before HPLC analysis. Waters 600 HPLC pump with a Waters 2489 UV/Visible detector was employed to separate the compounds through a reversed phase column (Thermo syncronis aQ, 4.6 mm × 250 mm, 5 μm) at a flow rate of 1 mL/min. The mobile phase consisted of acetonitrile (A) and 0.1% acetic acid in water (B). The gradient elution program was as follows: 10% A at 0 min and linearly up to 40% in 40 min, 90% at 41 to 50 min, and 10% at 51–65 min. The wavelength for UV detection was 354 nm and the column temperature was set at 25 °C. The compounds were identified by comparing with the retention time of standards, and quantified through calculating the area under the curve with external standards.

#### 2.4.4. Determination of Total Polysaccharide (TPS) content

The TPS content was determined with phenol–sulfuric acid method [39,40]. In brief, 80 mg sample was dissolved in 50 mL distilled water, and then the protein was removed with the savage method (sample solution:chloroform:n-butyl alcohol = 20:4:1). Then, four-fold amount of ethanol was added to the resultant viscous solution to precipitate the crude polysaccharide extract. The ethanol solution was centrifuged at 617.4 × g for 30 min to obtain the precipitate. The precipitate was washed successively with ethanol, acetone and ether, and dissolved in 250 mL distilled water. Then, 0.1 mL polysaccharide solution was mixed with 0.9 mL distilled water, 1 mL 5% phenol solution and 5 mL sulfuric acid in a test tube, and kept at room temperature for 30 min. The absorbance was measured at 490 nm with a UNICO-2012 UV-VIS spectrophotometer (Shanghai, China). A calibration curve of glucose was established, and the TPS content was determined from regression equation of calibration curve. The results were expressed as mg of glucose equivalents per mg of samples.

### 2.5. *In Vitro* Antioxidant Assays

The antioxidant capacity of OPD, OSK and OSD were detected by three methods, including 1-diphenyl-2-picrylhydrazyl (DPPH) scavenging, ferric reducing antioxidant power (FRAP) and reducing power, according to the literature [19,41] with slight modification. Trolox was used as a positive control, and the results were expressed as trolox equivalent antioxidant capacity.

As OPD was comprised of OSK and OSD, to illuminate the active constituents of okra pods, the concentration of OSK and OSD were used as OPD equivalent. According to the preliminary tests, the concentration ranges of OPD, ODS, OSK were 0.004–0.8, 0.0032–0.64 and 0.0008–0.16 mg/mL for DPPH assay; 1.0–4.0, 0.8–3.2 and 0.2–0.8 mg/mL for FRAP assay; and 0.1–4, 0.08–3.2 and 0.02–0.8 mg/mL for reducing power assay, respectively

For DPPH assay, sample solution (50 μL) with a proportional range (0.004–0.8 mg OPD/mL, 0.0032–0.64 mg OSK/mL and 0.0008–0.16 mg OSD/mL) was mixed with DPPH solution (100 μL, 1.28 × 10^−4^ mol/L) for measurement of free radical-scavenging activity (A1) and 95% ethanol (100 μL) for the control (A2). Distilled water (50 μL) was mixed with DPPH solution (100 μL) for the blank (A0). The absorbance was measured at 517 nm after the solutions were mixed and kept at room temperature for 30 min. The capacity to scavenge DPPH radical was calculated using the following equation:

(1) Scavenging activity (%) = [1 − (A1 − A2)/A0] × 100%.

The FRAP of three sample solutions (1.0–4.0 mg OPD/mL, 0.8–3.2 mg OSK/mL and 0.2–0.8 mg OSD/mL) were detected according to instruction of Beyotime Institute of Biotechnology. The diluted sample solution (5 μL) was mixed with FRAP working solution (180 μL) and kept for 5 min at 37 °C. The absorbance of the reaction mixture was then recorded at 593 nm. The standard curve was prepared using FeSO_4_, ranging from 0.15 to 1.5 mM.

In the reducing power determination, sample solution (0.5 mL for 0.1–4 mg OPD/mL, 0.08–3.2 mg OSK/mL and 0.02–0.8 mg OSD/mL) was mixed with the same volume of PBS (0.2 mol/L, pH 6.6) and potassium ferricyanide solution (1%, *w*/*v*), and incubation was carried out for 20 min at 50 °C. Trichloroacetic acid (0.5 mL, 10%, *w*/*v*) was added to the mixture, which was centrifuged at 201.6 × g for 10 min. After this, the upper layer of the solution (0.5 mL) was diluted with distilled water (0.5 mL) and then ferric chloride (0.1 mL, 0.1%, *w*/*v*) was added into it. The absorbance was measured at 700 nm.

### 2.6. Anti-fatigue Effects of OPD, OSK and OSD and Biochemical Analysis

Based on the same reason as the *in vitro* antioxidant tests, the doses of anti-fatigue of OPD, OSK and OSD were designed as OPD groups (0.75, 1.50 and 3.00 g/kg body weight), OSK groups (0.60, 1.20 and 2.40 g/kg body weight) and OSD groups (0.15, 0.30 and 0.60 g/kg body weight).

Mice were fed with basic diet in the experimental environment before the experiments. After 3 days adaptation, 120 mice were randomly divided into ten groups: control group (CG), 3 OPD, OSK, and OSD groups each. Each group had 12 mice. The control group (CG) was treated with sterile water, while the other groups were treated with corresponding extract. Water/extracts were administrated orally (8:00 a.m.) to mice once daily for 21 days, and the open field test and weight-loaded swimming test (WLST) were conducted on the 20th and the 21st days, respectively. After WLST, the mice were removed from the pool, dried with paper towels, and placed back in their original cages. The experimenters of WLST and open field test were blinded to the experimental conditions and animals were randomized before participation in experiments.

To elucidate the anti-fatigue effect of OSD during recovery, mice were anesthetized with ether and blood samples were collected from each group after WLST 24 h. Serum samples were obtained by centrifugation (896 × *g*, 15 min, 4 °C). Livers were quickly dissected and washed in ice-cold saline. All samples were frozen in liquid nitrogen and kept at −80 °C for further analysis.

#### 2.6.1. Open-Field Test

To verify the effects of OPD, OSK and OSD on locomotor activities, the mice were evaluated automatically using an open field computer-aided controlling system developed by us [42,43]. The apparatus consists of four metal tanks (30 cm in diameter, 40 cm in height) with a video camera fixed at the top. Experiments were performed in a quiet room, and the apparatus was illuminated by a light source of 120 Lux on the ceiling. One hour after dosing, each mouse was placed at the center of the metal tank and allowed to explore freely for 2 min and the distance travelled in 4 min, which is an index of locomotor activity, was calculated using software. Four mice were tested simultaneously.

#### 2.6.2. Weight-Loaded Swimming Test (WLST)

WLST was performed to induce fatigue according to the methodology described previously with slight modification [19,44,45]. One hour after dosing, the mice were loaded with lead sheets that were attached to the same position of their tails and weighed approximately 3% of their body weight. Then the weight-loaded mice were individually forced to swim in cylindrical recipients (diameter 40 cm), which were filled with water (25 ± 1 °C) to a depth of 30 cm. Exhaustion was classified as loss of coordinated movements and failure to rise to the surface within 10 s, and the swimming time was recorded immediately. The pool water was replaced after each session.

#### 2.6.3. Analysis of Biochemical Parameters Related to Fatigue

Serum was prepared for the determination of blood urea nitrogen (BUN) and blood lactic acid (BLA). Livers were prepared for the determination of malondialdehyde (MDA) equivalents, total superoxide dismutase (SOD), glutathione peroxidase (GSH-PX) and hepatic glycogen (HG). All biochemical parameters were determined using commercially available kits, and all procedures complied with the manufacturer’s instructions.

Briefly, the content of HG was determined using anthrone reagent at 620 nm, while BLA was determined using nitrotetrazolium blue chloride reagent at 530 nm. BUN was evaluated by urease with the glutamate dehydrogenase coupled enzyme method at 640 nm. GSH-PX activity was measured in a coupled system by measuring the decrease of triphosphopyridine nucleotide (NADPH) at 340 nm. The assay for SOD activity was based on its ability to inhibit the oxidation by O_2_ produced from the xanthine–xanthine oxidase system. One unit of SOD activity was defined as the amount that reduced the absorbance at 450 nm by 50%. The content of MDA equivalents was determined at 532 nm by reacting with TBA to form a stable chromophoric product.

### 2.7. Statistical Analysis

Chemical determinations were carried out in triplicate and results are expressed as mean values. The antioxidant assays were carried out in triplicate and the results were expressed as mean values ± standard deviations. Behavior and biochemical data were analyzed by SPSS statistical software (SPSS 19.0 Inc., Chicago, IL, USA). A one-way analysis of variance (One-way ANOVA) with Least-significant difference (LSD) test was used for inter-group comparison. P values less than 0.05 were considered statistically significant. The results were expressed as mean ± standard error of mean (SEM).

## 3. Results

### 3.1. Chemical Analysis of OSK and OSD

**Figure 1:**
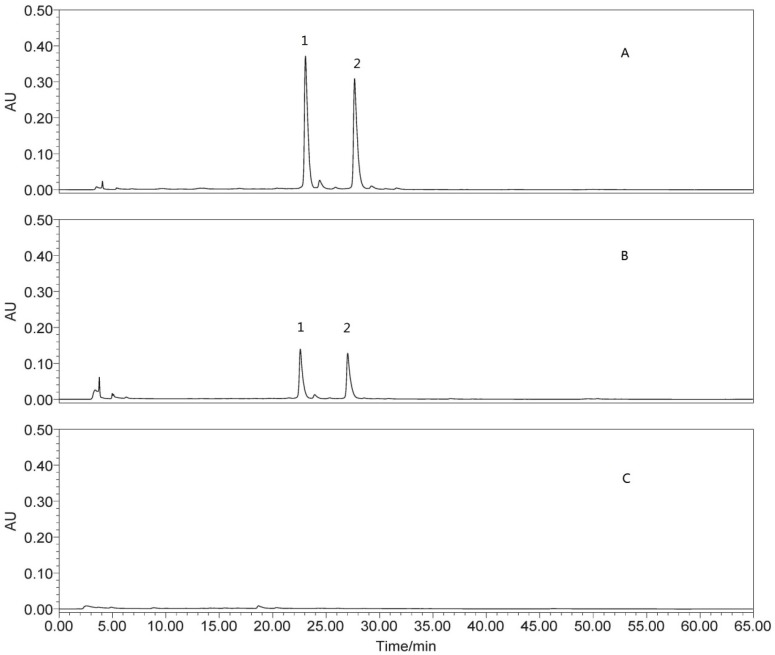
HPLC (High performance liquid chromatography) Chromatograms for okra seeds (OSD), pods (OPD) and skins (OSK): (**A**) chromatogram for OSD; (**B**) chromatogram for OPD; and (**C**) chromatogram for OSK; 1: quercetin-3-O-gentiobiose; and 2: isoquercitrin.

As shown in Table 1, the contents of TF and TP in OSD were much higher than those in OSK, meanwhile, the content of TPS in OSD was much lower than that in OSK. The compounds of isoquercitrin and quercetin-3-O-gentiobiose were only detected in OSD using HPLC (shown in Figure 1).

**Table 1:** Contents of total flavonoids (TF), total polyphenols (TP), total polysaccharides (TPS), isoquercitrin and quercetin-3-O-gentiobiose in okra skins (OSK), seeds (OSD) and pods (OPD).

Sample	OSD	OSK	OPD
TF (%)	5.35	~	1.02
TP (%)	29.5	1.25	6.73
TPS (%)	14.8	43.06	38.65
Isoquercitrin (%)	2.067	~	0.395
Quercetin-3-O-gentiobiose (%)	2.741	~	0.541

All values are the mean of triplicate measurements.

### 3.2. *In Vitro* Antioxidant Activity of OPD, OSK and OSD

As shown in Table 2, the antioxidant activity of OPD and OSD were proven by DPPH, FRAP and reducing power assays, and OSD showed much better antioxidant activity than OPD. However, OSK showed weak effect in reducing power assay and no effect in DPPH and FRAP tests. Therefore, OSD was the active part of okra pods for antioxidant activity.

**Table 2:** *In Vitro* antioxidant activities of okra pods (OPD), skins (OSK) and seeds (OSD).

	OSD (μmol of TE/g)	OSK *(μmol of TE/g)	OPD (μmol of TE/g)
DPPH	1585.48 ± 139.42	~	95.30 ± 12.02
FRAP	1429.80 ± 68.53	~	76.7 ± 5.32
Reducing power	2678.45 ± 148.55	77.98 ± 7.3	484.33 ± 29.05

Values are expressed as mean ± standard deviations, all the measurements were taken in triplicate; * OSK did not show antioxidant activity in 1-diphenyl-2-picrylhydrazyl (DPPH) and ferric reducing antioxidant power (FRAP).

### 3.3. Effects on Locomotor Activitiy of OPD, OSK and OSD

**Figure 2:**
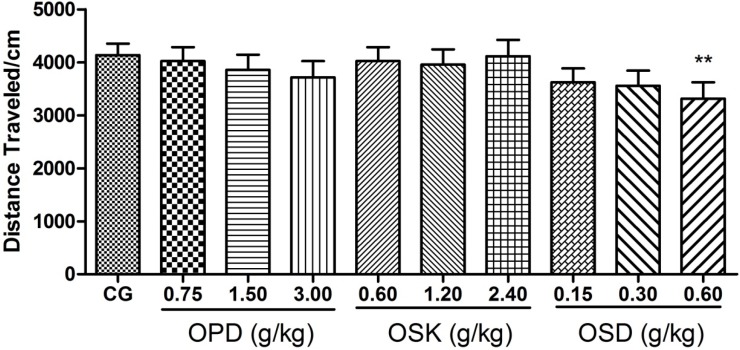
Effects of okra pods (OPD), skins (OSK) and seeds (OSD) on locomotor activities of mice. Values are expressed as mean ± standard error of mean (*n* = 10–12); ** *p* < 0.01 compared to control group.

Locomotor activities of mice were tested on the 20th day after being treated with OSD. As shown in Figure 2, high dose of OSD groups (0.6 g/kg) significantly reduced the total distance of the mice in the novel environment as compared with the control group (*p* = 0.04). However, no significant effects for other groups on the total distance were observed. These results showed that okra pods and seeds had no excitatory effect on the central nervous system. Moreover, it could be deduced that OSD might possess a slight sedative effect at a high dose (0.6 g/kg).

### 3.4. Effects on the Swimming Exhaustive Time of OPD, OSK and OSD (WLST)

**Figure 3:**
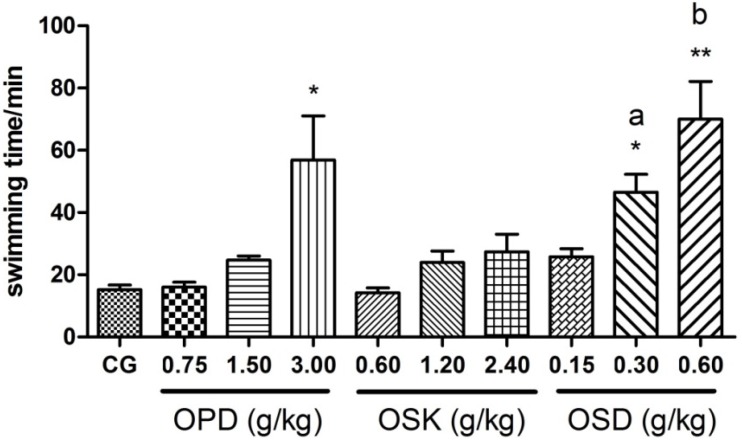
Effects of okra pods (OPD), skins (OSK) and seeds (OSD) on the weight-loaded swimming time of mice. Values are expressed as mean ± standard error of mean (*n* = 11–12); * *p* < 0.05 and ** *p* < 0.01 compared to control group; a: *p* < 0.05 compared to the low dose group; b: *p* < 0.05 compared to the middle dose group.

As shown in Figure 3, after supplement of OPD, OSK and OSD for 21 days, OPD (3 g/kg) and OSD (0.3 and 0.6 g/kg) treatment could markedly prolong the mice swimming time, which stretched up to 57.84 ± 12.37, 46.51 ± 5.82 and 70.05 ± 12.07 min (*p* < 0.05, *p* < 0.05 and *p* < 0.01), respectively, much longer than that in the control group (15.20 ± 1.49 min). Meanwhile, OPD (0.75 and 1.5 g/kg), OSD (0.15 g/kg) and OSK (0.6, 1.2 and 2.4 g/kg) treatment did not show significant increase of the swimming time in mice (14.73 ± 1.28, 23.96 ± 3.70 and 27.34 ± 5.72 min, respectively) as compared to the control group (*p* = 0.897, *p* = 0.398 and *p* = 0.268, respectively), but OSD (0.15 g/kg) showed a trend of increasing swimming time, but did not reveal a significant difference (*p* = 0.225). Moreover, One-way ANOVAs displayed significant differences on the swimming exhaustive time of the mice between groups (F (3, 40) = 12.839, *p* < 0.001). The swimming time of high dose of OSD group (0.6 g/kg) showed marked difference as compared to middle dose group (0.3 g/kg) (*p* = 0.019), and the same result was observed between 0.3-g/kg and 0.15-g/kg groups (*p* = 0.034), demonstrating OSD could prolong the exhaustive swimming time in a dose-dependent manner.

### 3.5. Effect of OSD on Biochemical Parameters in Mice after Weight-loaded Swimming Test

As the result of weight-loaded swimming test showed, OSD could markedly prolong the mice swimming exhaustive time; however, OSK treatment did not show significant increase of the swimming time in mice. Therefore, biochemical parameters were only determined on OSD treatment mice.

#### 3.5.1. Effect of OSD on BLA, BUN and HG

The difference of BLA, BUN and HG content among groups were significant (BLA: F (3, 40) = 8.257, *p* < 0.001; BUN: F (3, 40) = 8.596, *p* < 0.001; HG: F (3, 36) = 6.960, *p* < 0.001). As shown in Table 3, the levels of BLA and BUN in OSD groups were significantly lower than that in the control group 24 h after weight loaded swimming test (BLA: *p* < 0.05, *p* < 0.05 and *p* < 0.01 for 0.15, 0.3 and 0.6 g/kg, respectively; BUN: *p* < 0.01 for 0.15, 0.3 and 0.6 g/kg). The levels of HG in OSD groups were significantly higher as compared to control group (*p* < 0.05, *p* < 0.01 and *p* < 0.01 for 0.15, 0.3 and 0.6 g/kg, respectively).

**Table 3:** Effect of okra seeds (OSD) on blood lactic acid (BLA) and urea nitrogen (BUN) contents in serum and hepatic glycogen (HG) content in liver of mice after weight-loaded swimming test.

	Control Group	OSD Treated Groups		
	water	0.15 (g/kg)	0.3 (g/kg)	0.6 (g/kg)
**BLA (mmol/L)**	11.24 ± 0.4	9.76 ± 0.5 *	9.46 ± 0.5 *	8.13 ± 0.4 **
**BUN (mmol/L)**	10.72 ± 0.4	7.35 ± 0.3 **	7.13 ± 0.4 **	6.98 ± 0.3 **
**HG (mg/g liver)**	9.81 ± 0.9	17.3 ± 2.56 *	18.8 ± 2.43 **	14.76 ± 2.02 *

Values are expressed as mean ± standard error of mean (*n* = 10–12); * *p* < 0.05 and ** *p* < 0.01 compared with the control group in the same row.

#### 3.5.2. Effect of OSD on GSH-PX, MDA and SOD

The differences of SOD, GSH-PX activities and MDA equivalent among groups were significant (MDA: F (3, 40) = 8.355, *p* < 0.001; SOD: F (3, 42) = 9.876, *p* < 0.001; GSH-PX: F (3, 40) = 7.959, *p* < 0.001). As shown in Table 4, the MDA equivalent in OSD groups was significantly lower than that of control group after weight loaded swimming test (*p* < 0.01, *p* < 0.01 and *p* < 0.01 for 0.15, 0.3 and 0.6 g/kg, respectively). Furthermore, the levels of SOD and GSH-PX in OSD groups were significantly higher as compared to control group (SOD: *p* < 0.01, *p* < 0.01 and *P* < 0.01; GSH-PX: *p* < 0.05, *p* < 0.01 and *p* < 0.01 for 0.15, 0.3 and 0.6 g/kg, respectively).

**Table 4:** Effect of okra seeds (OSD) on malondialdehyde (MDA) content and glutathione peroxidase (GSH-PX), Superoxide Dismutase (SOD) activities in livers of mice after weight-loaded swimming test.

	Control Group	OSD Treated Groups		
	water	0.15 g/kg	0.3 g/kg	0.6 g/kg
**MDA (nmol/mg protein)**	0.772 ± 0.10	0.501 ± 0.02 **	0.487 ± 0.03 **	0.451 ± 0.03 **
**SOD (U/mg protein)**	113 ± 3.5	128 ± 4.2 **	137 ± 3.7 **	159 ± 4.0 **
**GSH-PX (U/mg protein)**	63.2 ± 3.6	80.4 ± 5.3 *	86.3 ± 4.8 **	98.5 ± 5.6 **

Values are expressed as mean ± standard error of mean (*n* = 10–12); * *p* < 0.05 and ** *p* < 0.01 compared with control group in the same row.

## 4. Discussion

Immature okra pod is a healthy vegetable that is consumed in most areas of the world. Previous studies have reported that immature okra pods had antioxidant and anti-fatigue effects [25,46], but its active constituents and potential mechanisms were not clear. In order to investigate the antioxidant and anti-fatigue activities of okra pods, we divided the fresh okra pods into seeds and skins, and obtained OSD, OSK and OPD through an extraction process, then the antioxidant activity *in vitro* of OSD, OSK and OPD was detected with DPPH, FRAP and reducing power and the anti-fatigue effect *in vivo* of OSD, OSK and OPD were studied using weight-loaded swimming test. Our results showed that OSD not only possessed a good antioxidant activity *in vitro*, but also significantly prolonged the swimming time of mice as compared to the control group, while, the OSK showed no effect in both. The exhaustive time of swimming is a direct measurement reflecting objectively the exercise endurance of the body [47], and the promotion of exercise endurance is correlated with longer swimming times. Therefore, the antioxidant and anti-fatigue part of OPD should be deduced as its seeds. Moreover, in this study, OSD (0.30, 0.60 g/kg) could markedly prolong the mice swimming time, and the human equivalent doses of okra seeds might be 0.9~1.8 g/kg (4.5~8.9 g/kg for okra pods) according to body surface area [48]. These doses can be reached by a human serving of the vegetable. However, the active constituents of okra are still unclear.

The results of chemical analysis in the present study showed that the content of polyphenols in OSD is about 24 times as much as that in OSK, but the content of polysaccharides in OSD is much lower than that in OSK (Table 1). Moreover, total flavonoids content and two compounds of isoquercitrin and quercetin-3-O-gentiobiose were only detected in OSD, and were not detected in OSK (Table 1). It has been proven that okra seeds contained epigallocatechin oligomers, catechin and its oligomers, isoquercitrin, quercetin-3-O-gentiobiose and other catechin and quercetin derivatives [27,28,29]. Furthermore, previous studies have reported that polyphenols like catechin and flavonoids like quercetin possess anti-fatigue activity due to their antioxidant activity [35,36,49,50,51]. Therefore, we deduced that polyphenols and flavonoids of OSD might be the antioxidant and anti-fatigue constituents. As for the reason that the effect of OPD was less than the effect of OSD, it might be that the high content of mucilaginous polysaccharides can affect the assimilation of polyphenols and flavonoids in OPD, which needs to be researched in the future.

When it comes to the anti-fatigue mechanism of OSD, an open-field test showed that OSD had no central nerve stimulation in mice, which proved that the anti-fatigue of OSD was not through central excitation. Apart from this, it was likely that OSD possessed a slight sedative effect in high doses (>0.6 g/kg). More data are needed to prove this because the small reduction in locomotion could also be the result of reduced motivation to explore or a possible anxiogenic effect. However, in this research, open-field test was used to exclude the possibility that OSD performed anti-fatigue activity by an excitatory effect on central nervous system, and more data to demonstrate the sedative effect of OSD did not present.

Furthermore, excessive reactive oxide species (ROS) produced during exhaustive exercise have been proven to be the major component of physical fatigue. Excessive ROS can affect the various metabolic and enzymatic processes by attacking polyunsaturated fatty acids to produce MDA and to lead to dysfunction of biofilms [13,52]. Because of this, ROS not only affect the enzymatic activity during energy supply and excitation-contraction coupling processes directly, but also accelerate the accumulation of waste including BLA and BUN. Moreover, the increasing level of BLA is an important cause of fatigue because of the lowering pH in tissues, and a higher content of BUN indicates lower exercise endurance. Therefore, it could not only enhance exercise endurance, but also alleviate physical fatigue and promote recovery by promoting the activity of enzymatic antioxidant systems including SOD and GSH-PX or directly supplement antioxidants. In the present study, biochemical parameters determination that were conducted 24 h after the swimming test indicates the OSD treatment significantly decreased the levels of BLA and BUN in blood, MDA in liver, and increased the levels of HG, SOD and GSH in liver during fatigue recovery, which proved that OSD could alleviate physical fatigue and promote recovery. Considering the results of antioxidant assays *in vitro* in our research, it could been deduced that OSD played the anti-fatigue effect not only via scavenging free radicals directly, but also by promoting the activities of antioxidant enzymes including SOD and GSH-PX.

## 5. Conclusions

In conclusion, the present study indicated that OSD is the anti-fatigue part of okra, and polyphenols and flavonoids were likely to be the active constituents of OSD because of their antioxidant activity. However, fatigue is a complex phenomenon, and the present study on the anti-fatigue mechanisms of okra is very preliminary. More details still need to be illuminated in the future.

## Supplementary Materials

Supplementary materials can be accessed at: http://www.mdpi.com/2072-6643/7/10/5435/s1.
