# Supplementary material for: Antioxidant and Anti-Fatigue Constituents of Okra
Source: Nutrients. 2015 Oct 26;7(10):8846–58. doi: 10.3390/nu7105435 (PMC4632455; doi:10.3390/nu7105435)
Supplement: Supplementary file 1 [file nutrients-07-05435-s001.docx]

**Supplementary Material**

Qualitative analysis of phenolic composition difference between okra seeds and skins

Materials and chemicals

The fresh okra pods were purchased from a market (Sanya, Hainan Province, China) in July, 2013. The plant was authenticated by Professor Bengang Zhang, the Institute of Medicinal Plant, Chinese Academy of Medical Sciences and Peking Union Medical College, Beijing, China, where the voucher specimens
(No. 20130705) have been deposited in Herbarium of the institute.

Chloride ferric (CP, Shanghai, China) is analytical grade and water was obtained from a Milli-Q water purification system (Millipore, MA, USA).

Methods

The fresh okra pods (50 g) were divided into skins (38.7 g) and seeds (10.6 g). And then, the skins and seeds were extracted with water (100 mL) under ultrasonic treatment for 30 minutes. Each filtered liquid was reacted with 1 mL chloride ferric solution (5%).

Results and discussion

Because phenolic constituents have a color reaction with chloride ferric solution, it is widely accepted that this reaction can be used to analyze phenols qualitatively. In this study, the extract of skins showed no reaction with chloride ferric solution, but the extract of seeds showed strong phenolic reaction. These results demonstrated that okra seeds contained much higher content of phenols than skins.

© 2015 by the authors; licensee MDPI, Basel, Switzerland. This article is an open access article distributed under the terms and conditions of the Creative Commons by Attribution (CC-BY) license (http://creativecommons.org/licenses/by/4.0/).
